# Dietary Influence on Growth, Physicochemical Stability, and Antimicrobial Mechanisms of Antimicrobial Peptides in Black Soldier Fly Larvae

**DOI:** 10.3390/insects15110872

**Published:** 2024-11-06

**Authors:** Shaojuan Liu, Muhammad Raheel Tariq, Qihui Zhang, Hui Wang, Fei Wang, Chaozhong Zheng, Kuntai Li, Zhikai Zhuang, Leiyu Wang

**Affiliations:** 1 Agricultural Products Processing Research Institute, Chinese Academy of Tropical Agricultural Sciences, Zhanjiang 524001, China; liushaojuan2023@163.com (S.L.); raheeltariq916@gmail.com (M.R.T.); realczzheng@163.com (C.Z.);; 2 College of Food Science and Technology, Huazhong Agricultural University, Wuhan 524088, China; 3 College of Food Science and Technology, Guangdong Ocean University, Zhanjiang 524088, China; 4 College of Biological Sciences and Engineering, Jiangxi Agricultural University, Nanchang 330045, China

**Keywords:** black soldier fly larvae, antimicrobial peptides, growth performance, physicochemical stability, transcriptomic analysis

## Abstract

Safe antibiotic substitutes are needed given the rise in antimicrobial resistance, environmental contamination, and stringent antibiotic regulations. Insect-derived antimicrobial peptides (AMPs) are promising candidates due to their antimicrobial activity, stability, and safety. This study investigates the antimicrobial mechanism of crude AMP extracts and their physicochemical characteristics in black soldier fly larvae (BSFL). The results indicated that BSFL reared on a wheat bran diet exhibited significantly improved growth performance and AMP production when compared to the other three diets. AMP extracts showed enhanced antimicrobial activity and physicochemical stability, including temperatures and metal ions except Cu^+^. Moreover, AMP extracts disrupted the cell membrane and inhibited the cell cycle of *Staphylococcus aureus* (*S. aureus*), thus exhibiting antimicrobial activity. Furthermore, transcriptomic and KEGG enrichment analyses identified 509 differentially expressed genes (DEGs) related to the Toll and IMD signaling pathways. STRING and GeneMANIA analyses confirmed the association of these pathways with immune response and AMP secretion. qRT-PCR results showed elevated expression of immune genes (*GNBP3*, *NFKBIA*, *GADD45*, and *Spz*) in BSFL following *S. aureus* immunization, consistent with RNA-seq findings. These findings offer a valuable reference for using AMPs as antibiotic substitutes in animal feeds and highlight the need for further research on AMP purification and the synergistic regulation of protein synthesis and AMP production in BSFL.

## Simple Summary

Due to the rise in antimicrobial resistance, environmental contamination, and stricter regulations on antibiotics, there is a growing need for safe alternatives. Insect-derived antimicrobial peptides (AMPs) are considered promising due to their antimicrobial activity, stability, and safety. In this study, we found that BSFL reared on a wheat bran diet showed significantly better growth and higher antimicrobial activity of AMP extracts compared to those on the other three diets. The AMP extracts demonstrated strong antimicrobial activity and stability against varying temperatures and metal ions. In addition, AMP extracts disrupted the cell membrane and inhibited the cell cycle of *Staphylococcus aureus* (*S. aureus*), contributing to their antimicrobial activity. Furthermore, we revealed that the immune response of BSFL and AMP secretion treated with *S. aureus* was associated with the Toll and IMD signaling pathways using the transcriptomic analysis, STRING, and GeneMANIA analysis and was confirmed by quantitative real-time PCR (qRT-PCR).

## 1. Introduction

With the increase in antimicrobial resistance, environmental pollution, and the stringent restrictions on the use of antibiotics, there is an urgent need to find antibiotic alternatives that are safe and environmentally friendly, addressing the growing concerns over antibiotic resistance and pollution [1]. Insect-derived AMPs are considered one of the potential alternatives to antibiotics due to their excellent antibacterial activity, stability, and safety [2,3,4,5], which are applied in the food and processing industries to reduce bacterial infection. Insect AMPs are mainly composed of small-molecule secondary metabolites, proteins, and peptides and widely defend against diverse microorganisms, including bacteria, fungi, viruses, and even tumor cells [6]. Insects have a complex immune system and develop immune functions depending on the innate immune system because of lacking adaptive immunity [7]. The insect immune system is composed of two main branches: cellular and humoral immunity, each playing a crucial role in defending against invasive pathogens [8]. Cellular immunity involves processes such as phagocytosis, nodulation, and encapsulation [8]. Phagocytosis refers to the engulfment and digestion of pathogens by immune cells, while nodulation and encapsulation involve the formation of multicellular structures around larger invaders, effectively isolating and neutralizing them [8]. Humoral immunity, on the other hand, is responsible for the production and secretion of AMPs and other immune factors like lysozyme into the hemolymph, the insect’s circulatory fluid [9,10]. This response is rapidly triggered upon pathogen invasion and is critical for neutralizing microbial threats [11]. The Toll pathway, primarily activated by fungi and Gram-positive bacteria, and the immune deficiency (IMD) pathway, which responds to Gram-negative bacteria, are two evolutionarily conserved signaling pathways that regulate this immune response [12]. These pathways play a pivotal role in controlling the expression of immune-related genes. Upon activation, they trigger the NF-κB signaling cascade, which leads to the upregulation of AMPs and other immune effectors, ensuring a rapid and effective defense against pathogens [13,14]. The biological significance of this dual-pathway system lies in its ability to tailor the immune response to the specific type of pathogen encountered, thereby optimizing the insect’s defensive capabilities [15]. The secretion of AMPs into the hemolymph constitutes the first line of defense in the insect humoral immune system, forming a critical barrier against microbial infections [16]. Studies on model organisms like *Drosophila* have provided extensive insights into the mechanisms of these pathways, highlighting their essential role in regulating immune gene expression and maintaining the insect’s overall health [17].

Black soldier fly larvae (BSFL), as a saprophagous insect fed on organic residues, have advantages of rapid growth and reproduction as well as excellent ability to transform agricultural or kitchen organic waste [18]. BSFL contains AMPs with stable and broad-spectrum antimicrobial properties [19]. Exposed to several conditions, such as immunization, diet influence, or temperature, BSFL can activate the innate immune system, thus producing AMPs in the fat body to defend against various pathogens. For example, ultraviolet irradiation (UV), often used in the food and processing industries to reduce bacterial infection, was one of the conditions tested [20]. Exposing AMPs to irradiation for different durations is often carried out to help in establishing appropriate storage conditions for the AMPs, ensuring that they retain their potency over time. Recent studies have focused on the AMPs of BSFL, including the antimicrobial activities, types, gene expression, and applications of AMPs [21,22,23,24]. Insect AMPs have two methods of action against pathogen bacteria: intracellular death and bacterial membranes [25,26]. AMPs have the ability to cause bacterial membranes to become porous or to interact with phospholipids through membrane solubilization, which can result in microscopic heterogeneity in lipid membranes and ultimately lead to the death of bacteria [27]. The action mechanisms of AMPs on bacterial membranes are divided into three categories: (1) the barrel-wall model, in which AMPs can penetrate bacterial membranes through the phospholipid bilayer by perforating the bacterial surface until it is completely ruptured [28]; (2) the ring-pore model, in which AMPs interact with the lipid head group, causing bilayer bending and vertical insertion into the membrane bilayer; and (3) the carpet model, in which AMPs cover the entire bacterial membrane [29], allowing the nonpolar side chain of the antimicrobial peptide to bind to aquatic organisms. In addition, AMPs can bind to specific enzymes in bacterial membranes or act on intracellular DNA and RNA to achieve the antimicrobial effects [17]. Additionally, AMPs can affect bacterial cells by interfering with metabolic processes and potentially influencing the cell cycle [30]. The cell cycle in *S. aureus* is crucial for developing targeted antimicrobial therapies. For example, antibiotics that target DNA replication (like quinolones) or cell wall synthesis (like β-lactams) interfere with specific stages of the cell cycle, thereby inhibiting bacterial growth and division [31]. As reviewed by a study that has illustrated the inhibition mechanism of a single AMP of BSFL [18], the AMPs expressed in vivo are multiple, and the inhibition mechanism of multiple AMP combinations in defense bacteria has not been studied yet. Moreover, it has been reported that the secretion of AMPs is diet-dependent and influenced by the rearing environment, which could modulate the intestinal microbiota composition of BSFL and then secrete immune effectors and molecules [32]. Another study shows the interaction between two immune genes, *Duox* and *TLR3* [33], and the result only showed that these two genes could regulate key intestinal microorganisms to achieve the result of pathogen inhibition. However, the regulatory mechanisms are not fully understood, but currently few articles concern this issue.

In this study, to explore the antimicrobial mechanism of AMP extracts and its physicochemical characteristics in BSFL under different diets, we first investigated the growth performance and antimicrobial activity of AMP extracts by feeding four different diets and the stability of AMP extracts by evaluating the antimicrobial activity under different physicochemical conditions. Moreover, we examined the cell morphology and cell cycle of *S. aureus* to explore the antimicrobial mechanism of AMP extracts against pathogenic bacteria. Furthermore, the analysis of transcriptomic data, STRING and GeneMANIA, and the qRT-PCR experiment were conducted to reveal the antimicrobial mechanism of AMP extracts in BSFL. This work revealed the growth performance and antimicrobial activity of AMP extracts under different diets, together with the physicochemical stability under different conditions and the antimicrobial mechanism of AMPs related to the Toll and IMD signaling pathways, which was not only established as a novel reference for the antimicrobial mechanism of AMPs but also provided theoretical support for substituting for the antibiotics in animal feeds.

## 2. Materials and Methods

### 2.1. BSFL Feeding and Treatments

The BSFL used in this study were obtained from Nanjing, Jiangsu. Four diets were purchased from Huainan, Anhui. The BSFL were reared at 25 °C and 65% humidity in a climate room. After hatching at wheat bran for three days, the BSFL were randomly divided into four groups (1000 larvae per group) and fed with the following diets on 70% moisture: Group A, 0.50 kg of wheat bran; Group B, 0.25 kg of wheat bran mixed with 0.25 kg of soybean cake powder; Group C, 0.25 kg of wheat bran mixed with 0.25 kg of peanut cake powder; Group D, 0.25 kg of wheat bran mixed with 0.25 kg of rapeseed cake powder. The diets were selected based on their practical use in BSFL rearing and included wheat bran, a common and nutritionally balanced feed, and mixtures with soybean, peanut, or rapeseed cake powders. The larva was reared in boxes with 2000 cm^3^ (length: 24.5 cm, width: 16 cm, height: 8 cm). The feeding was carried out at once during the whole larval stage and lasted 8 days. After four days, 10 larvae were recorded for larval length, and 100 larvae were taken for larval weight measurement. After the whole experiment, 10 g of larvae (wet weight) were used for the determination of water content, crude fat, and crude protein content.

### 2.2. Immune Hemolymph Collection

The pathogenic bacterium *Staphylococcus aureus* (*S. aureus*) was preserved in our laboratory and maintained on an LB agar medium (tryptone 10 g/L, yeast extract 5 g/L, NaCl 10 g/L, agar 20 g/L, pH 7.20). For the inducement of AMPs, 10 μL *S. aureus* bacterial solution (10^7^ cells) was injected into BSFL by using a fine needle (diameter 0.26 mm, length 9 mm), and the larvae were kept at 25 °C for 24 h under starvation. The larvae were washed with sterilized water. To prevent melanization, the 1.5 mL collection tube contained the phenylthiourea with 1 μg/mL and the protease inhibitor with 10 μg/mL when the insect hemolymph was collected. First, the cephalic region of larvae was cut with sterile scissors. Next, the residual larvae were squeezed to collect the hemolymph, and then the immune hemolymph was rapidly collected in an ice-cold tube for 30 s to inhibit the activation of prophenoloxidase. Subsequently, the collected hemolymph was centrifuged at 4 °C and 12,000× *g* for 35 min to remove hemocytes and cell debris, and the supernatant was stored at −80 °C for further analysis. The supernatant was recovered and freeze-dried in a −50 °C freeze dryer (lypholization). The resulting freeze-dried powder was the crude AMP extract and was stored at −20 °C.

### 2.3. Ultraviolet Irradiation Treatment of AMPs

At room temperature, the AMP extracts were exposed to 30 W ultraviolet (UV) irradiation for 15 min, 30 min, 1 h, 2 h, and 4 h, respectively. *S. aureus* was used as an indicator of bacterium. The antimicrobial activity of AMP extracts was determined by filter paper diffusion assay and evaluated by the inhibitory zone diameter [34]. The untreated AMP extracts were used as a positive control group (n = 3).

### 2.4. Metal Ion Treatment of AMPs

The metal ion concentration of 10 mmol/L was prepared from FeCl_3_, MgCl_2_, CuCl_2_, CaCl_2_, BaCl_2,_ and MnCl_2_ with distilled water, respectively. Moreover, 10 μL of the AMP extracts were mixed in a ratio of 1:1 with the above metal ion solution to obtain the final 5 mmol/L concentration of each metal ion. The sample was mixed 1:1 with distilled water as the control group and placed at 37 °C for 3 h to determine the antimicrobial activity. Each treatment was performed using three replicate samples.

### 2.5. NaCl Treatment of AMPs

Aliquots of 20 μL of the AMP extracts were added with 6%, 8%, 10%, and 12% NaCl, respectively, and fully shaken until dissolved to the final concentrations of 3%, 4%, 5%, and 6% NaCl solutions. The samples added with an equal volume of distilled water were used as the control group. All samples were placed at 37 °C for 2 h to determine the antimicrobial activity. Each treatment was performed using three replicate samples.

### 2.6. Surfactant Treatment of AMPs

The surfactants, including Tween-20, Tween-40, Tween-60, Tween-80, sodium dodecyl sulfate (SDS), and urea, were prepared into 10% (*v/v*) solution with sterile water and were mixed 1:1 with the AMP extract solution. After incubation for 2 h, the antimicrobial activity was measured. Each treatment was performed using three replicate samples.

### 2.7. Storage Time Treatment of AMPs

Storage temperatures play a crucial role in the use and preservation of the AMP extracts. The AMP extracts were placed at −80 °C, −20 °C, and 4 °C, and their antimicrobial activities were determined on days 0, 10, 30, and 60. Each treatment was performed using three replicate samples.

### 2.8. Thermal Treatment of AMPs

The AMP extracts were placed in the water bath at 40 °C, 60 °C, 80 °C, and 100 °C and incubated at 121 °C in an autoclave for 20 min for thermal stability determination, respectively. After exposure to the above treatments, the antimicrobial activity of the AMP extracts against *S. aureus* was determined using the filter paper diffusion assay. The untreated AMP extracts were used as a positive control group (n = 3).

### 2.9. Antimicrobial Activity Assay

To evaluate the antimicrobial activity of the AMP extracts against Gram-positive *S. aureus*, the diameter of the inhibition zone was examined by a radial diffusion assay. *S. aureus* was picked from the plate and inoculated into the 250 mL shake flask containing 50 mL of LB medium. The shake flask was shaken at 200 rpm overnight at 37 °C. The culture broth of *S. aureus* (being adjusted to 1 × 10^8^ CFU/mL) was added to the LB plates. The filter paper was sterilized and socked in the crude extract of AMPs at 37 °C for 18–24 h. The inhibitory zone diameter was measured to evaluate the antimicrobial activity of AMPs. The experiment was repeated three times.

### 2.10. Cell Membrane Permeability and Morphology Scanning

To explore the antimicrobial mechanism of the AMP extracts against *S. aureus*, the morphology of *S. aureus* after treatment with AMP extracts was visualized by fluorescence microscope (FM), scanning electron microscopy (SEM), and transmission electron microscopy (TEM), respectively. The *S. aureus* was inoculated into LB medium and cultivated at 37 °C on a rotary shaker at 120 rpm for 10 h. Subsequently, each culture broth was added with 1× minimal inhibitory concentrations (MIC) and 2× MIC AMP extracts and followed by cultivation at 37 °C and 120 rpm for 4 h. The control was cultivated in the absence of AMP extracts. The MIC was determined by using serial dilutions of the AMP extracts. Each dilution was inoculated with a standard concentration of *S. aureus* (e.g., 1 × 10^8^ CFU/mL) and incubated at 37 °C for 18–24 h. The lowest concentration of the AMP extracts that prevented visible bacterial growth was recorded as the MIC [35].

PI (propidium iodide) staining was used to examine cell membrane permeability as part of the investigation into the antimicrobial mechanism of AMP extracts against *S. aureus*. Bacterial cells were collected by centrifugation at 6000× *g* for 5 min and then washed with 0.1 M PBS three times. For FM observation (Nikon Eclipse NI-SS, Tokyo, Japan), the bacterial cells were added 50 μg/mL propidium iodide (PI) for 10 min and centrifuged at 4 °C and 6000× *g* for 5 min, and then washed with 0.1 M PBS two times to remove excessive PI. Samples determined by SEM were prepared by the method of the previous study [36] and observed using Hitachi SU8010. TEM samples were prepared according to the literature of [37] and observed on Hitachi H-7060.

### 2.11. Flow Cytometry Detection of S. aureus Cell Cycle

To investigate the antimicrobial mechanism of AMP extracts against *S. aureus*, the cell cycle progression of *S. aureus* cells was assessed using flow cytometric analysis. After cultivation with LB medium at 37 °C and 120 rpm for 10 h, the culture broth of *S. aureus* was added to 1× MIC and 2× MIC AMP extracts and cultivated for 4 h. The final culture broth was centrifuged at 2500× *g* for 5 min to collect *S. aureus* cells, and then the cells were washed with 0.1 M sterile PBS three times. Subsequently, the *S. aureus* cells were fixed with 70% PBS alcohol solutions and kept at 4 °C overnight, and then washed with 0.1 M PBS two times. Then, the cells were added with RNA enzyme and incubated at 37 °C for two hours, followed by PI dyeing for 30 min. Finally, the samples were tested by flow cytometer. The control group was performed as described above, but without AMP extracts. Attune Nxt 1260 Infinity Ⅱ was used as an indicator to evaluate the effects of AMP extracts on the plasma membrane of *S. aureus* cells stained by Attune Nxt 1260 Infinity Ⅱ. A fluorescence dye PI can penetrate the damaged cell membrane and intercalate into a nucleic acid.

### 2.12. Total RNA Extraction

According to the previous experiments, the BSFL reared on wheat bran was used for immunization and total RNA extraction. Firstly, the unimmunized and immunized larvae were crushed separately with liquid nitrogen. Then, the total RNA was extracted from the tissue using TRIzol^®^ Reagent according to the manufacturer’s instructions (Invitrogen, Carlsbad, CA, USA). Moreover, 200 µL chloroform was added to the above tissue homogenates, and tubes were vortexed for 15 s. After setting for 2 min, the supernatant was collected in a 1.5 mL tube after centrifugation at 12,000× *g* for 15 min at 4 °C. Then, 500 µL of isopropanol was added to the supernatant and gently mixed the liquid well. After 10 min, the supernatant was removed after centrifugation at 12,000× *g* for 10 min. Moreover, 1 mL of 75% ethanol was added to the retained pellet. Similarly, the sediment was kept after centrifugation at 7500× *g* for 5 min for repeating twice. The collected RNA pellet was air-dried on a clean bench for 10 to 15 min. Next, 50 µL of RNase-free water was added to dissolve the RNA pellet. Finally, RNA concentration was measured using NanoDrop OneTM (Thermo Fisher Scientific Inc., Valencia, CA, USA). The extracted RNA was stored at −80 °C for subsequent transcriptome sequencing.

### 2.13. RNA Sequencing

A qualified RNA sample was used for library construction and sequencing. Firstly, Oligo (dT) with magnetic beads was matched and paired to the A-T bases with the ployA of mRNA that the mRNA was isolated from total RNA. Then, mRNA was added to fragmentation buffer and randomly broken into small fragments about 300 bp to achieve fragmented mRNA. Random primers, reverse transcriptase, and the fragmented mRNA used as a template were reversed into a cDNA strand. The cDNA with stable structure was formed after two-strand synthesis. Thirdly, End-Repair Mix was added to the synthesized cDNA to complete the sticky end of double-stranded cDNA into a flat end and then a single. A base was added to the 3′ end for the Y-shaped connector. Finally, the products were purified and segmented. PCR amplification was performed using the sorted product to obtain the final library that was quantified by the QuantiFluor^®^ dsDNA System (Illumina, San Diego, CA, USA) and then sequenced by proportional mixing.

Transcriptome sequencing was carried out on an Illumina HiSeqxten/NovaSeq 6000 (Illumina, San Diego, CA, USA) sequencer using paired-end (2 × 150 bp) read technology. To obtain high-quality sequencing data to ensure the accuracy of subsequent analysis, the raw paired-end reads were filtered, trimmed, and qualified by SeqPrep (https://github.com/jstjohn/SeqPrep accessed on 1 May 2023) and Sickle (https://github.com/najoshi/sickle accessed on 8 May 2023) with default parameters to remove the connector sequences, low-quality reads, sequences with a high rate of N (N represents uncertain base information), and excessively short sequences. Then, clean reads were separately aligned to the reference genome of *Hermetia illucens* (No. PRJEB37575) with orientation mode using HISAT2 software (v2.2.1) (http://ccb.jhu.edu/software/hisat2/index.shtml accessed on 6 June 2023) [38]. The mapped reads of each sample were assembled by StringTie (https://ccb.jhu.edu/software/stringtie/index.shtml?t=example accessed on 15 June 2023) in a reference-based approach [39].

To identify the DEGs of BSFL AMPs under immunization, the expression level of each transcript was calculated and normalized according to the transcripts per million reads (TPM) method. RSEM (http://deweylab.biostat.wisc.edu/rsem/ accessed on 8 July 2023) was used to quantify gene abundances [40]. Essentially, differential expression analysis was performed using DESeq2 [41] with the parameters Q value ≤ 0.05 and DEGs with |log2FC| > 1 and Q value ≤ 0.05. GO (Gene Ontology) and KEGG (Kyoto Encyclopedia of Genes and Genomes) analyses were performed at a Bonferroni-corrected *p*-value ≤ 0.05 compared with the whole-transcriptome background. The GO functional enrichment and KEGG pathway analysis were analyzed by Goatools (https://github.com/tanghaibao/Goatools accessed on 15 July 2023) and KOBAS (http://kobas.cbi.pku.edu.cn/home.do accessed on 15 July 2023) [42]. A Fisher exact test was used for GO enrichment and KEGG pathway analysis, in which the corrected *p*-value (p_FDR) less than 0.05 was considered a significant enrichment.

### 2.14. Protein–Protein Interaction (PPI) Analysis

To further explore the protein interactions of DEGs in BSFL under immunization, we constructed the protein network of DEGs by STRING database analysis [43]. The protein network of up-regulated DEGs was exhibited with connected nodes and a high confidence of 0.700. The protein network of up-regulated DEGs in the Toll and IMD signaling pathways was conducted using a GeneMANIA database analysis [44].

### 2.15. Real-Time Quantitative PCR (qRT-PCR) Analysis

Up-regulated DEGs were randomly selected for qRT-PCR to validate the transcriptome sequencing data reliability. Total RNA was reverse transcribed to cDNA using the PrimeScript II 1st Strand cDNA Synthesis Kit (TaKaRa) kit. The steps of reverse transcription (RT) are as follows: The RT reaction solution was prepared on ice and consisted of 5× PrimeScript RT Master Mix (Perfect Real Time) with 2 μL, 1 μL of total RNA, and 7 μL of sterile enzyme-free water. The RT reaction solution was gently mixed and incubated at 37 °C for 15 min for reverse transcription reaction. Then, it was placed at 85 °C for 5 s to inactivate reverse transcriptase, and finally the cDNA was obtained for qRT-PCR. Subsequently, a qRT-PCR experiment was conducted using the SYBR Premix Ex TaqTM II (TliRNaseH Plus) (TaKaRa, Shiga, Japan) as the fluorescent dye. PCR amplifications were performed in 20 μL reaction mixtures containing 10 μL 2× TB Green Premix Ex Taq™ (Tli RNaseH Plus) (TaKaRa, Shiga, Japan), 0.8 μL PCR Forward Primer (10 μM), 0.8 μL PCR Reverse Primer (10 μM), 0.4 μL ROX Reference Dye Ⅱ (50×), 2 μL cDNA, and 6 μL RNase-Free dH_2_O. The PCR cycling conditions proceeded as follows: 95 °C for 5 min, 40 cycles of 95 °C for 15 s, and 60 °C for 30 s. Melting curve analysis confirmed the specificity of the amplification. The qRT-PCR reaction was performed using the ABI 7500/7500 Fast Real-Time PCR System, and the 40 s ribosomal protein gene (RPS4e) was selected as the internal reference gene [32]. Three technical and three biological replicates of each reaction were performed, and the relative expression of the genes was calculated using the 2^−ΔΔCt^ algorithm. The specific primers designed using NCBI are shown in Table S1.

### 2.16. Statistical Analysis

GraphPad Prism 8.0 and DPS v7.05 software were used for curve fitting and statistical analysis, and each experiment was performed in triplicate (n = 3). The results were reported as mean ± standard deviation (SD) and compared by one-way analysis of variance (ANOVA) with Duncan’s multiple tests and *p*-value < 0.05 was considered statistically significant. The evolutionary trees and heatmap were visualized on the image processing website Chiplot (www.chiplot.online accessed on 10 September 2023), and the data were analyzed on the online platform Majorbio Cloud Platform (www.majorbio.com accessed on 30 September 2023).

## 3. Results

### 3.1. Effects of Different Diets on the BSFL Growth Performance, Components, and Antimicrobial Activity of AMPs

**Figure 1:**
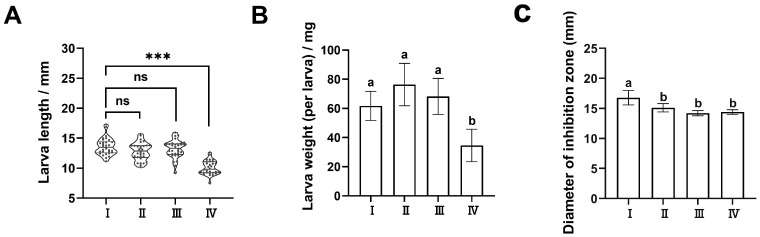
The growth performance and antimicrobial activity of AMP extracts under different diets. I: pure wheat bran cultivation; II: soybean cake flour and wheat flour 1:1 cultivation; III: peanut cake flour and wheat bran 1:1 cultivation; IV: rapeseed cake flour and wheat bran 1:1 cultivation. (**A**) The length of BSFL in four different diets. *p*-values were determined using Student’s *t*-test. *** *p* < 0.001 means significant difference. ns means no significant difference. (**B**) The weight of per larva. (**C**) The diameter of the inhibition zone of AMP extracts in four different diets. The letters of a and b within a column without a common superscript letter mean significant difference (*p* < 0.05). The results were reported as mean ± standard deviation (SD) and compared by one-way analysis of variance (ANOVA) with Duncan’s multiple tests (*p* < 0.05).

Feeding substrates influence the growth performance and composition of larvae and their immune systems. In this study, the components and growth performance of BSFL and the antimicrobial activity of AMP extracts under four diets (A: wheat bran; B: wheat bran: soybean cake powder = 1:1; C: wheat bran: peanut cake powder = 1:1; D: wheat bran: rapeseed cake powder = 1:1) were shown in Figure 1 and Table 1, respectively. The length and weight of BSFL showed a significant difference among the four diets (*p* < 0.05), of which the length and weight in diet A was the highest when compared to diet D (Figure 1A,B). Similarly, the results showed that the antimicrobial activity of AMPs against *S. aureus* in diet A was the strongest when compared to the other three diets (Figure 1C), which was consistent with the results of BSFL growth. In the subsequent experiments, we used wheat bran as a feeding diet for rearing BSFL. Additionally, the components of BSFL, including crude protein, crude fat, and moisture content, exhibited significance among the four diets (Table 1), suggesting that feeding substrates could affect the composition of BSFL.

### 3.2. Stability of AMPs Under Different Physicochemical Conditions

**Figure 2:**
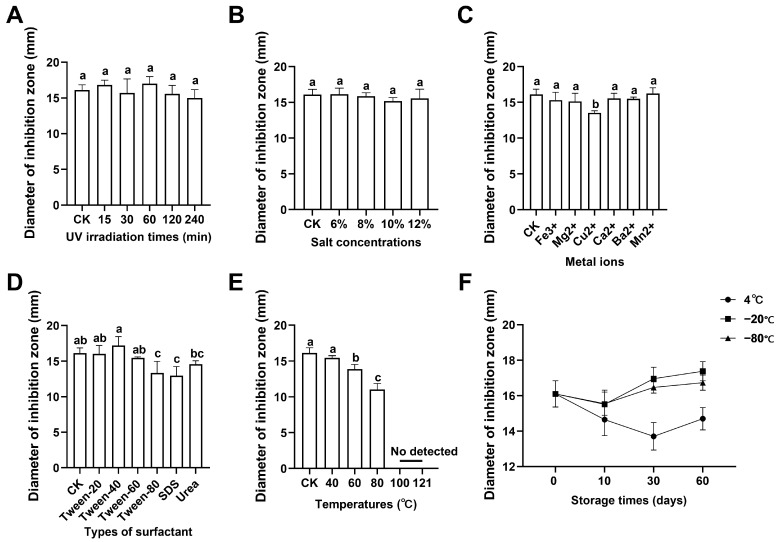
Stability of the AMP extracts from BSFL under different physicochemical conditions. (**A**–**F**) were the stability of the AMP extracts under different UV irradiation times, salt concentrations, metal ions, surfactants, temperatures, and storage temperatures for 10, 30, and 60 days, respectively, and expressed by the diameter of the inhibition zone. The results were reported as mean ± standard deviation (SD) and compared by one-way analysis of variance (ANOVA) with Duncan’s multiple tests. The letters of a–c means within a column without a common letter differ in (**B**,**C**) (*p* < 0.05).

To investigate the stability of AMP extracts extracted from the larvae and cultured with a single wheat bran diet, the following analysis was studied. UV irradiation and salt concentration had no effect on the antimicrobial activity of AMPs (Figure 2A,B). In addition, the antimicrobial activity of AMP extracts incubated with Cu^2+^ for a period showed a significant decrease compared to the other five different metal ions (Figure 2C). The antimicrobial activity of AMP extracts treated with surfactants Tween-80 and SDS was significantly inhibited (Figure 2D). Moreover, it found that the antimicrobial activity of AMP extracts decreased with the increase in temperatures at 40 °C, 60 °C, 80 °C, 100 °C, and 121 °C for 30 min, and the AMP extracts still retained antimicrobial activity at 80 °C, indicating that the AMP extracts have thermal stability (Figure 2E). Moreover, it was observed that the antimicrobial activity of AMPs stored at 4 °C had decreased gradually but increased at −20 °C and −80 °C, suggesting that lower temperature and longer time storage had a benefit to the stability of AMP extracts (Figure 2F).

### 3.3. Cell Membrane Permeability of S. aureus Treated with AMPs

**Figure 3:**
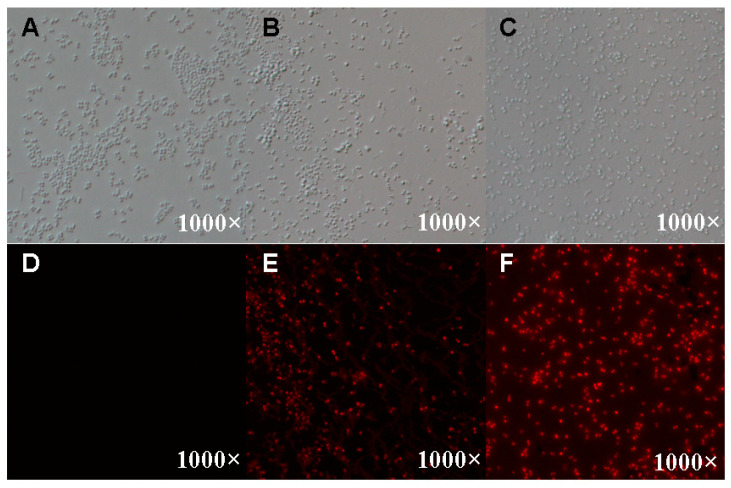
Cell membrane permeability of *S. aureus* treated with different concentrations of AMP extracts. (**A**–**C**) separately showed the optical microscopic observation of *S. aureus* without AMP extracts or with AMP extracts at MIC concentration and 2MIC concentration, respectively, while (**D**–**F**) respectively exhibited the fluorescence microscope observation of *S. aureus* stained by PI of different concentrations of AMP extracts.

PI (propidium iodide) staining was used to examine cell membrane permeability as part of the investigation into the antimicrobial mechanism of AMPs against *S. aureus*. PI cannot pass through live cell membranes, but it can penetrate damaged cell membranes and stain nuclei. A stronger fluorescence intensity indicates a stronger membrane permeability. Our results showed that the *S. aureus* treated with the AMP extracts stimulated a strong red fluorescence under green laser and was dose-dependent for AMP extract concentration compared to the control group by FM observation (Figure 3), indicating that the AMP extracts could destroy the cell membrane structure and further increase the cell membrane permeability of *S. aureus*.

**Figure 4:**
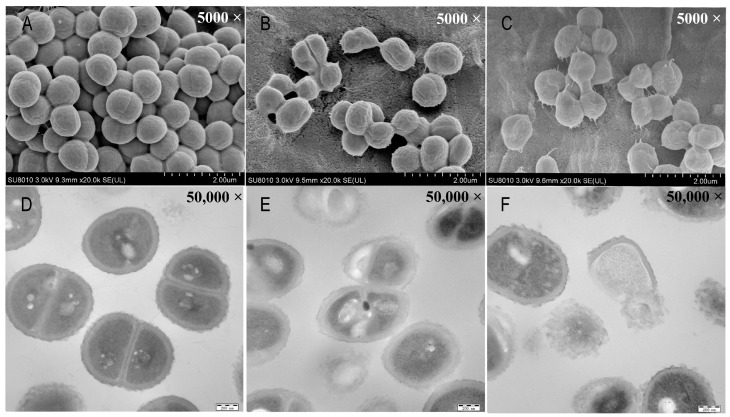
The morphology of *S. aureus* with different concentrations of AMPs under two electron microscopes. (**A**–**C**) were the observations of *S. aureus* without AMPs or with AMPs at MIC concentration and 2MIC concentration by scanning electron microscope, respectively. (**D**–**F**) individually represented the images of *S. aureus* under different concentrations of AMPs by transmission electron microscope. The scale bars were 2 μm in figure (**A**–**C**), and 200 nm of scale bars was used in figure (**D**–**F**).

SEM and TEM were used to observe the cell morphology and integrity of *S. aureus*. The SEM images showed that the *S. aureus* in the control group exhibited a smooth surface and intact cell morphology (Figure 4A). However, the surface and cell morphology of *S. aureus* were wrinkled and adhered after exposure to MIC concentrations of AMP extracts (Figure 4B) and more obvious under the treatment with two MIC concentrations of AMP extracts (Figure 4C). Compared to the intact intracellular structures in the control group via TEM observation (Figure 4D), AMP extracts caused obvious damage to the cell structure of *S. aureus* in a dose-dependent manner and resulted in cell breakage and cytoplasm leakages (Figure 4E,F). This result indicated that the mode of action of AMP extracts against *S. aureus* might target the membrane lesions of *S. aureus*.

### 3.4. Cell Cycle Arrest in S. aureus Treated with AMPs

**Figure 5:**
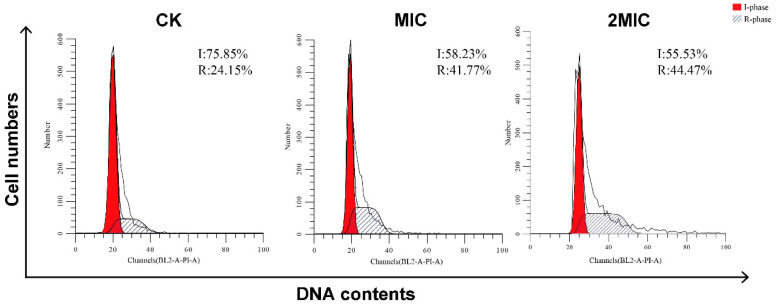
The cell cycle of *S. aureus* treated with AMP extracts. There were successively the cell cycles of *S. aureus* without AMP extracts or with AMP extracts at MIC concentration and 2MIC concentration.

To investigate the antimicrobial mechanism of AMPs against *S. aureus*, the cell cycle progression of *S. aureus* cells was assessed using flow cytometric analysis. The two broad cell cycle phases are interphase (I phase) and mitosis (R phase). Of which, interphase contains the processes of cell growth, DNA replication, and preparation for division. The process of cells dividing and forming two new daughter cells is called mitosis. This study observed that the AMP extracts led to cell accumulation of *S. aureus* in R-phase in a dose-dependent manner (Figure 5). In detail, the population of *S. aureus* cells in the R-phase increased from 24.14% in the control group to 41.77% at 1× MIC concentration of AMP extracts and 44.47% at 2× MIC concentration of AMP extracts. Meanwhile, the percentage of *S. aureus* cells in I-phase presented a negative relation to the concentrations of AMP extracts, respectively. The above results suggest that the AMP extracts would induce an appreciable cell cycle arrest of *S. aureus* cells in the R phase and inhibit DNA replication to resist pathogen invasion.

### 3.5. Transcriptomic Analysis of the BSFL After S. aureus Immunization

**Figure 6:**
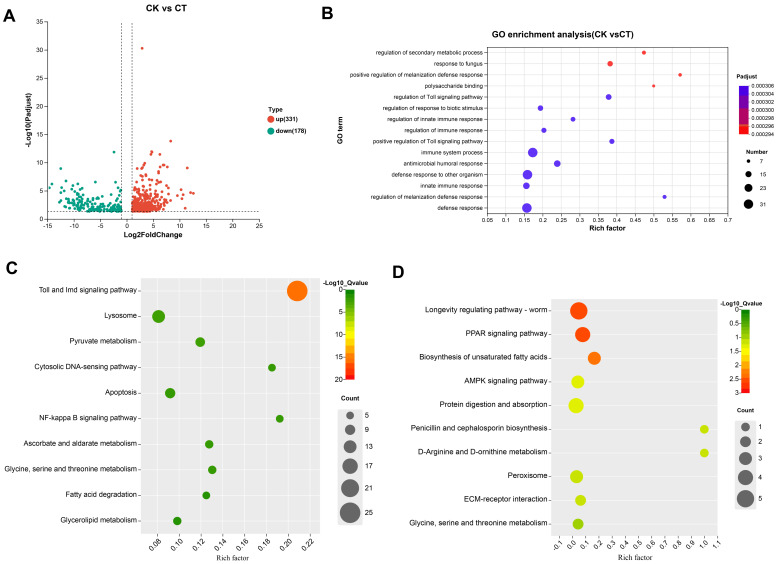
Transcriptomic analysis of differential gene expression of BSFL under *S. aureus* immunization. (**A**) The volcano plots displayed a total of 509 DEGs. The red and green plots represented 331 up-regulated genes and 178 down-regulated genes, respectively. (**B**) GO enrichment and analysis of DEGs of BSFL under immunization. (**C**) The top 10 KEGG pathways are enriched in the up-regulated genes. (**D**) The top 10 KEGG pathways are enriched in the down-regulated genes.

To explore the antimicrobial mechanism of AMPs, transcriptome data were analyzed, and the results showed that a total of 509 DEGs were identified between the control group and the immunized BSFL, accounting for 3.67% of the total unigenes. Among the 509 DEGs, 331 genes were significantly up-regulated and 178 genes down-regulated (Figure 6A). The results of GO enrichment indicated that the numbers of DEGs were assigned to the top 15 GO enrichment terms in molecular function, cellular component, and biological process reached 140, 104, and 211, respectively. As the secondary metabolite, the induction and biosynthesis of AMPs are closely related to the host immune defense against infection. Compared to the control group, the DEGs significantly up-regulated in immunized BSFL were involved in terms of immune response (e.g., regulation of innate immune response, immune system process, regulation of immune, and antimicrobial humoral immune response). In addition, these up-regulated DEGs were related to the signaling pathway (regulation of the Toll signaling pathway and positive regulation of the Toll signaling pathway), defense response (positive regulation of the melanization defense response), and regulation of secondary metabolic processes (Figure 6B).

**Figure 7:**
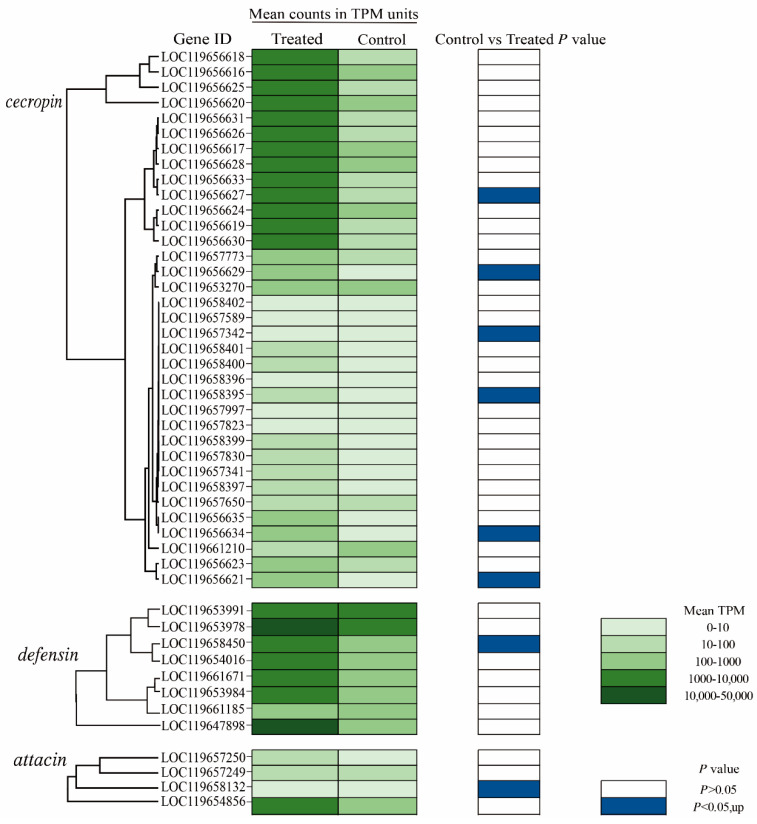
The evolutionary tree and average expression level of the AMP gene family in the immunization of *S. aureus* with BSFL by RNA-seq analysis. The average expression level of TPM units is displayed in green scale, and the statistical difference between the blank group and the treatment group is displayed in blue (up).

To determine the metabolic pathways of DEGs of the BSFL under immunization, KEGG enrichment analysis showed that the up-regulated DEGs were remarkably associated with immune-related pathways, including Toll and IMD signaling pathways and the NF-kappa B signaling pathway (Figure 6C and Table S2), which played an important role in a range of immune responses. AMPs belong to the typical small-molecule peptides consisting of amino acid residues. Most significantly, the down-regulated DEGs in the immunized BSFL were also related to the endopeptidase activity and serine-type endopeptidase activity, indicating that the AMPs could effectively avoid the degradation of endopeptidase (Figure 6D). Moreover, it presented that the down-regulated DEGs were enriched in the pathways of fatty acid metabolism, such as the longevity-regulatory pathway, PPAR signaling pathway, biosynthesis of unsaturated fat, and AMPK signaling pathway (Figure 6D and Table S3). It was also involved in protein digestion, absorption, and amino acid metabolism, which all may be related to the synthesis and stability of AMPs in vivo. Noteworthy, the *cecropin*, *defensin*, and *attacin* genes are important components of the AMP gene family. In this study, 35 cecropin, 9 defensin, and 4 attacin genes were annotated. The abundance of transcripts ranged from 10 to 20,000 TPM. The results indicated that the gene expression of AMPs treated with *S. aureus* was obviously higher than the control group (Figure 7), suggesting that the AMPs developed an important pathway of immune response.

### 3.6. Protein–Protein Interaction Analysis of DEGs of the BSFL Under S. aureus Immunization

**Figure 8:**
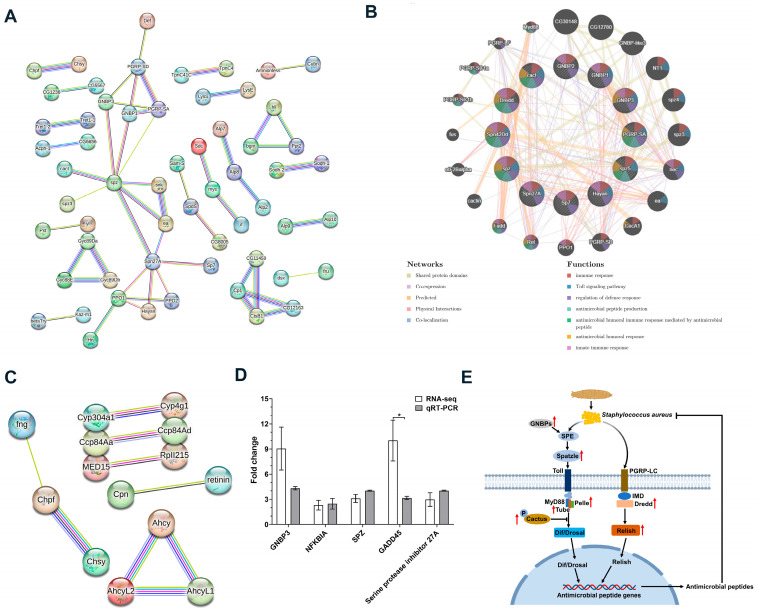
Analysis of up-regulated and down-regulated DEGs by protein–protein interaction analysis. (**A**) Schematic of the prediction of up-regulated DEGs by STRING database analysis (confidence, 0.700). (**B**) GeneMANIA database analysis of up-regulated DEGs involved in the Toll and IMD signaling pathways. (**C**) STRING database analysis of down-regulated DEGs. (**D**) The transcriptional expression of DEGs in the Toll and IMD signaling pathways by RNA-seq and qRT-PCR (n = 3). *p*-values were determined using Student’s *t*-test. * *p* < 0.05 means significant difference. (**E**) The diagram of AMPs against *S. aureus* by the Toll and IMD pathways.

To further investigate the mechanism of AMP production, PPI analysis exhibited that a total of 136 genes from 331 up-regulated DEGs and 37 genes from 178 down-regulated DEGs of BSFL under *S. aureus* immunization was annotated by *Drosophila melanogaster* (RSEM v1.1.17) as a reference genome. To explore the protein interactions of DEGs, the protein network of DEGs was constructed by the STRING tool. The PPI network of up-regulated DEGs with high confidence was displayed in Figure 8A. Interestingly, we observed that the relationship of up-regulated DEGs was relevant to the thiamine metabolism, folate biosynthesis, metabolic pathways, lysosome, and Toll and IMD signaling pathways (Table S4). To further investigate the interaction of up-regulated DEGs in the Toll and IMD signaling pathways, we employed the GeneMANIA tool to predict the PPI network and functional relationship among the selected genes, including *GNBP1*, *GNBP2*, *GNBP3*, *Hayan*, *Dredd*, *Sp7*, *Spz5*, *PGRP-SA*, *Spz*, *Cactus*, *Spn42Dd*, and *Spn27A* (Figure 8B). The result of GeneMANIA analysis showed that the up-regulated DEGs were associated with the immune response and the antimicrobial peptide production (Table S5), which was in accordance with the results of RNA-seq and STRING analysis. Simultaneously, we also conducted the PPI network analysis on down-regulated DEGs and discovered that it was involved in the process of cuticle development, sulfur compound metabolic process, and chitin-based cuticle development (Figure 8C and Table S6).

In the light of the above results, we further detected the expression of *GNBP3*, *NFKBIA*, *Spz*, *GADD45*, and *serine protein inhibitor 27A* genes related to the Toll and IMD signaling pathway and immune response by RT-qPCR. The result indicated that the mRNA expression of these genes was consistent with the result of RNA-seq (Figure 8D and Table S7). Collectively, we illustrated the pathway of AMP extracts acting on *S. aureus* in the Toll and IMD pathways and AMP production in Figure 8E. The diagram exhibits that when *S. aureus* attacks BSFL, the activated *Spz* gene is combined with toll receptors to activate the expression of the *Pelle* gene. Meanwhile, *S. aureus* stimulates the expression of the *Dredd* gene through the IMD pathway, thus activating *Relish* and then stimulating the expression of antibacterial peptide genes by the Toll and IMD pathways to develop an immune response.

## 4. Discussion

BSF is one of the most promising insects that could convert a variety of organic waste, including food scraps and animal feces from various sources, into protein resources or other nutrient substances [45,46,47]. Understanding the factors that influence the health and immune response of BSF larvae is critical for optimizing their use in these applications. While the current study primarily focused on the immune response of BSF larvae to pathogen exposure, the effect of diet on their immune system warrants further investigation. Feeding substrates can affect the growth performance and components of BSFL [32,48]. Nutrition plays a vital role in the development and function of the immune system in insects, including BSF larvae [32]. Dietary components such as proteins, lipids, carbohydrates, vitamins, and minerals are essential for supporting various physiological processes, including the production of AMPs and the activation of immune-related signaling pathways [49,50,51]. Despite the growing interest in BSF as a model organism, there is a paucity of research specifically examining the dietary effects on the immune response of these larvae. In this study, the growth performance of BSFL was significantly better when fed a diet of wheat bran compared to a combination of wheat bran and rapeseed cake flour. The larvae on the wheat bran diet showed increased length and weight, indicating that growth performance is closely associated with the type of feeding substrate. This difference in growth may be due to the presence of certain anti-nutritional factors in rapeseed cake flour, which is potentially toxic and could negatively affect the larvae’s growth. Additionally, the antimicrobial activity of AMP extracts was higher in larvae fed the wheat bran diet. These results demonstrated that the growth performance and components of BSFL (Table 1) and the immunity of AMP extracts were associated with feeding substrates, which was consistent with previous studies [32,52]. However, one limitation of our study is that we did not analyze the specific nutritional composition of the diets or investigate how individual nutrients might affect immune function and AMP production. Wheat bran may provide a balanced array of nutrients or contain bioactive compounds that support optimal larval growth and immune function. Previous studies have suggested that overall diet quality and nutrient balance can significantly impact insect physiology and immunity [53,54]. While high protein or fat content can influence immune responses, factors such as nutrient digestibility, the presence of anti-nutritional factors, and nutrient synergy also play crucial roles [55,56]. Therefore, we cannot definitively attribute the enhanced growth and AMP activity observed in larvae fed wheat bran to specific dietary components.

Insect AMPs have good antimicrobial activities, stability, and safety and are considered a good substitute for antibiotics [57,58]. Exposure to ultraviolet (UV) light has been shown to impact various aspects of insect physiology, including immune function. UV radiation can induce stress responses and affect the expression of genes involved in immunity [59]. Ultraviolet irradiation is often used in the food processing industries to reduce bacterial infection. In this study, we detected the antimicrobial activity of AMP extracts under different physicochemical conditions to evaluate the stability of AMP extracts. The results showed that UV irradiation and NaCl concentrations had no effect on the antimicrobial activity of AMP extracts, but Cu^2+^ and the surfactants of Tween-80 and SDS had a significant inhibition. In addition, the antimicrobial activity of AMP extracts decreased with the increase in temperatures at 40 °C, 60 °C, 80 °C, 100 °C, and 121 °C for 30 min, while AMP extracts still retained antimicrobial activity at 80 °C, indicating that the AMP extracts have thermal stability (Figure 2E). Moreover, it was observed that the antimicrobial activity of AMP extracts stored at 4 °C had decreased gradually but increased at −20 °C and −80 °C, suggesting that lower temperature and longer time storage had the benefit to the stability of AMP extracts (Figure 2F). These results may suggest that while the AMP extracts are thermally stable, their activity could be compromised by factors associated with long-term storage at lower temperatures, such as changes in protein conformation, interactions with other components in the hemolymph, or the gradual degradation of bioactive compounds. It has been reported that insects could regulate a low body temperature to defend against bacterial infection by upregulating immune functions such as heat-shock proteins and AMPs [60]. Temperature is a critical factor affecting insect development and metabolism. BSFL have specific temperature ranges within which they thrive, and deviations from these ranges can impact their growth and immune function [61]. Temperature could not only affect protein activity and then regulate antimicrobial activity but also play an important role in feed processing. In our study, AMPs had great thermal tolerance that played an important role in immune response and were of great popularization value as feed additives. Similarly, many studies have reported that BSFL has antibiotic resistance and metal resistance [62,63,64]. Ions, particularly those of trace elements like zinc, copper, and iron, play vital roles in immune function and overall health in insects [65]. These elements are essential for the proper functioning of enzymes involved in the immune response, such as those responsible for producing AMPs [66]. A study has evidenced that cadmium and catechol in food waste contamination have no deleterious impacts on physiological performance and bioconversion efficiency in BSFL [63]. Moreover, BSFL could effectively decrease the content of heavy metals and change the intestinal microorganisms when treated with landfill leachate [47]. The activity of AMP extracts is known to be influenced by the presence of metal ions such as Mg^2^⁺ and Ca^2^⁺, which can either stabilize or destabilize their structure and function. Previous studies have shown that certain metal ions can enhance or inhibit the antimicrobial efficacy of AMPs depending on the specific ion and concentration involved [67,68]. In contrast to these findings, the present study observed that the antimicrobial activity of AMP extracts was not significantly affected by most metal ions tested, except for Cu^2^⁺, which led to a marked decrease in activity. In the presence of Cu^2^⁺, there might be competition for binding sites on AMPs. If Cu^2^⁺ binds preferentially to these sites, it could prevent other essential metal ions from interacting with AMPs, thus affecting their function [69]. This competition could explain why AMP extracts show reduced activity specifically in the presence of Cu^2^⁺. This discrepancy highlights the complexity of AMP interactions with environmental factors and suggests that the specific composition of the hemolymph extract and the conditions of the study may have contributed to these unique findings. Collectively, AMP extracts had great stability under different conditions such as UV, temperatures, surfactants, and heavy metal ions. The stability of AMP extracts under UV irradiation and varying temperatures suggests that the peptides possess robust structural features. While our study did not explore how diet might influence the physicochemical properties of AMPs, it is possible that the nutritional status of the larvae affects the expression and post-translational modifications of AMPs, thereby influencing their stability [70]. Future research could investigate the relationship between diet, AMP structure, and stability under environmental stressors.

There are two mechanisms of insect AMPs against pathogen bacteria, including bacterial membranes and intracellular death. To investigate the antimicrobial mechanism of AMP extracts derived from BSFL, cell membrane permeability, cell morphology, and cell cycle were examined by PI staining, SEM, TEM, and flow cytometry. The results exhibited that AMPs could inhibit bacterial growth in two ways, including disrupting or altering the cell membrane of *S. aureus* or blocking the cell cycle of bacteria. When AMPs disrupt the structure and permeability of the cell membrane, PI dyes could enter the cells, bind to nucleic acids, and show red fluorescence under green laser light. In this study, the results of SEM and TEM showed that the AMP extracts could disrupt the integrity of *S. aureus* and change cell surface morphology, leading to cell lysis and efflux. In addition, flow cytometry analysis indicated that the AMP extracts had a blocking effect on the cell cycle of *S. aureus*, leading to cell cycle arrest, which presented a dose-dependent manner of AMP concentration and was similar to the previous results [71].

To explore the mechanism of antimicrobial activity of AMP extracts, we determined the transcriptomic analysis, and the results revealed that a total of 48 antimicrobial peptide genes were expressed, which was in accordance with the result of transcriptome annotation [72]. In our results, the DEGs significantly up-regulated in immunized BSFL were involved in terms of immune response, Toll signaling pathway, and positive regulation of Toll signaling pathway and positive regulation of melanization defense response. Studies have indicated that the melanization process, which involves the synthesis of melanin to encapsulate and kill foreign entities, is often positively regulated during an immune challenge [73,74,75]. This aligns with the mention of “positive regulation of melanization defense response” in the current study. The Toll signaling pathway is a well-known component of the innate immune system in insects, which is responsible for recognizing pathogens and initiating an immune response [76]. Previous research has shown that components of the Toll pathway, such as the recognition molecules and downstream effectors, are up-regulated upon infection or immunization, supporting the current observation in BSFL [77]. It has been reported that the DEGs of *Drosophila* under immunization were involved in the Toll and IMD pathways [17]. Similarly, our study showed that the KEGG enrichment analysis of DEGs was mainly enriched in the Toll and IMD pathways when BSFL were subjected to bacterial solution stress. In this regard, we further explored the PPI network of DEGs by STRING and geneMANIA database analysis. All of the results of the PPI network and functional enrichment implied that the up-regulated DEGs were associated with Toll and IMD pathways and AMP production. These results showed that the production and antimicrobial effects of AMPs were mainly regulated by the Toll and IMD signaling pathways, which provided preliminary evidence for the mechanism of AMP production in BSFL. Moreover, this result could provide a basis for further research on the mechanism of AMPs. For example, the specific mechanisms by which this pathway regulates the production of AMPs, the role of upstream immune factors, and the identification of their molecular targets remain areas for further investigation. Moreover, the PPI analysis of down-regulated DEGs revealed an association with chitin-based cuticle development. Chitin, a key structural component of the fungal cell wall, plays a crucial role in maintaining fungal cell integrity and flexibility [78]. Fungal cell wall is considered a great drug target because its integrity is essential for cell survival [79]. Chitin is a crucial component of the insect exoskeleton and plays a vital role in maintaining cuticle integrity [80]. In our study, the observed down-regulation of genes involved in chitin synthesis is more likely associated with the immunization method (injection) and the subsequent wound healing process in BSFL. The physical injury from injection can trigger wound healing responses, and the expression of some genes involved in chitin synthesis and cuticle repair may be temporarily suppressed or modulated after 24 h as part of the normal healing process [81,82]. This down-regulation may not directly relate to the antimicrobial mechanisms of AMPs against S. aureus but reflects the dynamic changes in gene expression associated with tissue repair and immune modulation following injury. Further research is needed to elucidate the specific roles of these genes in wound healing and their interaction with immune responses in BSFL.

In order to verify the transcriptomic results, we selected five key up-regulated genes from the GO enrichment and KEGG significant enrichment pathways. The up-regulated genes such as *GNBP3* (gene-LOC119649581), *NFKBIA* (gene-LOC119647312), and *Spz* (gene-LOC119657145) genes mainly played immune signal transduction roles in the Toll signaling pathway [10,83,84]. The synthetic gene *GADD45* (Gene-loc119652163) was involved in apoptosis and the NF-κB signaling pathway, and the gene expression was low in normal cells, but the protein expression was rapidly enhanced under stress response, especially suffering from DNA damage [85]. It has an immune synergistic effect in response to the injection of the bacterial solution. Serine protease inhibitor 27A (gene-LOC119649801) could decrease the activity of serine peptidase inhibitors. In our study, the gene expression tendencies of these genes in qRT-PCR and RNA-seq data were up-regulated and consistent despite the differences in gene expression values, indicating that the results based on RNA-seq were reliable. The process of BSFL acting on *S. aureus* in the Toll and IMD pathways and AMP production was described as follows: *S. aureus,* as a representative Gram-positive bacterium, invaded BSFL, activating the Toll signaling pathway, and then induced the serine protease cascade, resulting in the cleavage of the polypeptide expressed by *spaetzle*. Next, the sheared *spätzle* as a ligand activated the Toll receptor [86,87], and then the downstream effector factors were aggregated, respectively, such as *MyD88*, *Tube,* and *Pelle*, and further induced Cactus protein ubiquitination and phosphorylation. Finally, the nuclear key factor *Dif/Dorsal* had access to the nucleus and initiated the expression of antimicrobial peptide genes. Moreover, both the *Pelle* and *Cactus* genes have an obvious regulation trend, consistent with the signaling pathway produced by the dipteran model organism Drosophila in response to Gram-positive bacteria [17]. Only Gram-negative bacteria can induce the IMD pathway [88]. However, we observed that *S. aureus* could activate the IMD signaling pathway by stimulating *Dredd* in this study. *Dredd* is a protease associated with apoptosis and an essential enzyme in the IMD signaling pathway [89]. *Relish*, activated by the Dredd gene, is an NF-κB precursor protein and is inhibited in the cytoplasm by the IκB-like IKK domain in inactivated cells [90]. Once the IMD signaling pathway is activated, Relish is cleaved to deliver the N-terminal RHD ed into the nucleus, driving the high expression of antimicrobial peptide genes. Likewise, the *GADD45* synthetic gene involves apoptosis and the NF-kappa B signaling pathway, which participates in the DNA repair process and has immune synergy in response to bacterial injections. Collectively, in our study, the gene expression of *Pelle*, *Cactus*, *Dredd*, *Relish,* and *GADD45* in RNA-seq data was significantly enriched in the Toll and IMD signaling pathways under *S. aureus* immunization, which was consistent with the results of previous studies [91,92].

One limitation of our study is the relatively small number of biological replicates (n = 3) used in the experiments. While this sample size provided initial insights into the antimicrobial activity and stability of AMP extracts, we recognize that it may not capture the full extent of biological variability. Future studies with larger sample sizes will be necessary to confirm these findings and to strengthen the statistical power of the results.

## 5. Conclusions

This study demonstrates the potential of BSFL-derived crude AMP extract as a viable alternative to traditional antibiotics. The results highlight that the diet of BSFL, particularly wheat bran, significantly influences their growth performance and antimicrobial efficacy. Our findings highlight the potential of wheat bran as a high-performing diet for BSFL, leading to enhanced growth and antimicrobial activity. However, further studies are needed to dissect how individual dietary components influence immune function and AMP production in BSFL. Understanding these mechanisms could facilitate the development of optimized diets to enhance the antimicrobial potential of BSFL. In addition, AMP extracts exhibited robust stability under various physicochemical conditions, such as temperature and metal ion presence, making them suitable for practical applications. Furthermore, AMP extracts effectively disrupted the cell membrane and inhibited the cell cycle of *S. aureus*, underscoring its potent antimicrobial activity. Transcriptomic analysis provided deeper insights into the molecular mechanisms governing the secretion of AMPs, revealing the key roles of the Toll and IMD signaling pathways. This study’s findings suggest that diet not only impacts the growth of BSFL but also enhances the production of AMPs in the hemolymph. These results offer a foundation for further exploration into optimizing AMP production and application, especially in developing antibiotic alternatives for use in animal feed. In conclusion, BSFL-derived AMPs hold a significant promise as a stable, effective antimicrobial agent. This study contributes to our understanding of how dietary factors and immune pathways can be manipulated to enhance AMP production, providing a basis for future research aimed at harnessing AMPs for practical and commercial use.

## Figures and Tables

**Table 1:** Effects of different diets on dry weight crude protein, crude fat, and moisture content in black soldier fly larvae.

Class	Crude Protein/%	Crude Fat/%	Moisture Content/%
A	54.91 ± 0.72 ^b^	11.58 ± 0.27 ^b^	78.10 ± 1.07 ^a^
B	59.40 ± 2.86 ^a^	14.10 ± 0.33 ^a^	69.71 ± 3.00 ^b^
C	54.16 ± 0.94 ^b^	14.83 ± 0.21 ^a^	62.64 ± 2.21 ^c^
D	48.54 ± 0.84 ^c^	4.87 ± 0.76 ^c^	70.28 ± 4.40 ^b^

Note: A: pure wheat bran cultivation; B: soybean cake flour and wheat flour 1:1 cultivation; C: peanut cake flour and wheat bran 1:1 cultivation; D: rapeseed cake flour and wheat bran 1:1 cultivation. The results were reported as mean ± standard deviation (SD) and compared by one-way analysis of variance (ANOVA) with Duncan’s multiple tests. The letters of a–c within a column without a common superscript letter mean significant difference (*p* < 0.05).

## Data Availability

The original contributions presented in this study are included in the Supplementary Materials; further inquiries can be directed to the corresponding authors upon reasonable request.

## Supplementary Materials

The following supporting information can be downloaded at: https://www.mdpi.com/article/10.3390/insects15110872/s1, Table S1. The sequences of primers of real-time quantitative PCR; Table S2. KEGG enrichment of up-regulated DEGs by RNA-seq analysis; Table S3. KEGG enrichment of down-regulated DEGs by RNA-seq analysis; Table S4. KEGG enrichment of up-regulated DEGs of BSFL under immunization by String analysis; Table S5. Functional analysis of up-regulated DEGs in the Toll and IMD signaling pathway by GeneMANIA analysis; Table S6. GO enrichment of down-regulated DEGs of BSFL under immunization by String analysis; Table S7. The relative mRNA expression of DEGs involved in the Toll and IMD signalling pathway by RNA-seq and qRT-PCR (n = 3).

## Abbreviations

Antimicrobial peptides (AMPs); black soldier fly larvae (BSFL); *Staphylococcus aureus* (*S. aureus*); minimal inhibitory concentrations (MIC); differentially expressed genes (DEGs); fluorescence microscope (FM); scanning electron microscopy (SEM); transmission electron microscopy (TEM); ultraviolet (UV); sodium dodecyl sulfate (SDS); propidium iodide (PI); transcripts per million reads (TPM); protein–protein interaction (PPI); real-time quantitative PCR (qRT-PCR); 40 s ribosomal protein gene (RPS4e); reverse transcription (RT); standard deviation (SD); analysis of variance (ANOVA); interphase (I phase); mitosis (R phase).
